# Screening and Analysis of Antifungal Strains *Bacillus subtilis* JF-4 and *B. amylum* JF-5 for the Biological Control of Fusarium Wilt of Banana

**DOI:** 10.3390/jof9090886

**Published:** 2023-08-29

**Authors:** Yajie Duan, Zhencai Pang, Shunli Yin, Weijun Xiao, Huigang Hu, Jianghui Xie

**Affiliations:** 1 Key Laboratory of Tropical Fruit Biology, Ministry of Agriculture and Rural Affairs, South Subtropical Crops Research Institute, Chinese Academy of Tropical Agricultural Sciences, Zhanjiang 524091, China; 2 Key Laboratory of Hainan Province for Postharvest Physiology and Technology of Tropical Horticultural Products, South Subtropical Crops Research Institute, Chinese Academy of Tropical Agricultural Sciences, Zhanjiang 524091, China; 3 College of Tropical Crops, Hainan University, Haikou 570228, China; 4 College of Agriculture and Life Sciences, Kunming University, Kunming 650214, China

**Keywords:** Fusarium wilt of banana, antagonistic strain, inhibition mechanism, biocontrol application, secondary metabolite, *Bacillus*

## Abstract

Purpose: This study aimed to identify the antagonistic bacteria from the rhizosphere of healthy bananas that can effectively suppress the Fusarium wilt of banana, and to further investigate the inhibitory mechanism. Method: The primary and secondary screening techniques were implemented using the double-plate and fermentation antagonism methods. The strain was identified based on physiological and biochemical tests, *16S rRNA* gene sequencing, and specific gene amplification. The effects of crude extract on the protein content, lipid peroxidation, and pectinase activity of mycelia were determined from the identified isolates. Results: Two antagonistic bacteria, JF-4 and JF-5, were screened and initially identified as *Bacillus subtilis* (GenBank: OR125631) and *B*. *amylum* (GenBank: OR125632). The greenhouse experiment showed that the biological control efficiency of the two antagonists against the Fusarium wilt of banana was 48.3% and 40.3%, respectively. The catalase content produced by lipid peroxidation increased significantly after treatment with the crude extracts of JF-4 and JF-5 at concentrations of 0.69 μmol/L and 0.59 μmol/L, respectively. The protein and ergosterol content and pectinase activity decreased significantly. The two antagonistic bacteria might inhibit the growth of pathogens by enhancing lipid peroxidation and decreasing the synthesis of cell metabolites. Twenty compounds were identified by gas chromatography–mass spectrometry (GC-MS). *B. subtilis* JF-4 was further sequenced and assembled to obtain a complete circular chromosome genome of 681,804,824 bp. The genome consisted of a 4,310,825-bp-long scaffold. Conclusion: The findings of this study may help elucidate the mechanism behind this biocontrol isolate.

## 1. Introduction

Banana (*Musa* spp.) is a valuable primary agricultural product worldwide. With its remarkable yield, it serves as a staple food for about 400 million people worldwide. It ranks as the fourth-largest food crop after rice, wheat, and corn by the World Food and Agriculture Organization. However, the occurrence and spread of Fusarium wilt pose the most serious threat and challenge with the rapid development of the global banana industry [1]. *Fusarium oxysporum* f. sp. *cubense* (*Foc*) is a devastating soil-borne disease that destroys vascular bundles and kills banana plants [2]. It is also one of the most serious fungal diseases in the world. Banana withered wilt disease is the main factor limiting banana production and the bottleneck in banana-industry development because of the current unavailability of any effective control agent. Biological control is recognized as a relatively safe and effective prevention and control method [3]. It is mainly accomplished using biocontrol microorganisms or microbial metabolites for plant disease control. Its high selectivity, easy degradation of residues, good environmental compatibility, human and livestock safety, and other advantages have attracted the attention of researchers [4]. Many studies have reported the screening of biocontrol bacteria that prevent and control the occurrence of Fusarium wilt, including *Actinomycetes*, *Bacillus*, *Pseudomonas*, *Trichoderma*, and nonpathogenic *F*. *oxysporum* [5]. Studies investigating the prevention and control of banana Fusarium wilt started relatively late in China. The updated information on the existing biological control technology is limited. Some strains often appear mutated and degraded, suggesting the need for a constant supply of new biocontrol strains with high efficiency [6]. This study aimed to use healthy banana roots in banana gardens to screen highly effective biocontrol strains in intersoiled ground and enrich the existing pool of biocontrol strains. This study may provide the basis for effective control of banana Fusarium wilt.

The biological control mechanism mainly includes the induction of competition, antagonism, and resistance [7]. Antagonism is the main mechanism of biocontrol. The cell-wall-degrading enzymes, such as glucanase, are the main antagonistic substances produced by biocontrol bacteria [8]. The antimicrobial protein produced by the metabolism of *Bacillus subtilis* B25 increases, distorts, or deforms the size of hyphae or spores of *F*. *oxysporum*, revealing its inside contents clearly, thus inhibiting the growth and reproduction of the pathogen. A few studies reported the toxicological effects of these inhibitory mechanisms [9]. Nigris et al. (2018) showed that the antifungal components of the endophytic *B*. *subtilis* bound to the lecithin of the cell membrane of pathogens and formed a complex that caused membrane perforation, thus destroying the plasma membrane of *F*. *vulgare* [10]. Li et al. (2022) found that biocontrol bacteria metabolizing bacteriostatic substances effectively destroyed the cell membrane of the pathogen and affected its metabolic activities [8]. Surfactin is a cyclic lipopeptide that inhibits the growth of bacteria, viruses, and mycoplasma and acts as a biosurfactant [8]. Pinkas et al. (2020) found that *B. subtilis* produced a cyclic lipoheptapeptide, which was a surface-active antimicrobial agent [11]. The antibacterial activity of cyclolipopeptides was mainly manifested in their ability to interact with the cell membrane and destroy its permeability, which could change the surface tension of the cell membrane, form pores, and eventually trigger cell apoptosis [7,8,9].

Metabolomics can reflect the influence of microorganisms caused by genetic and environmental changes. Therefore, the environmental adaptability of microorganisms or the utilization degree of different substances can be explored using metabolomics [11,12,13]. Awla et al. (2016) isolated a strain of *Streptomyces oryzae* with anti-blast activity and detected the antifungal compounds of the extract against oryzae oryzae using GC-MS and LC-MS [1]. Microorganisms such as actinomycetes and microfungi produce many secondary metabolites with various biological activities. Metabolomics research focuses on studying metabolite differences caused by changes in temporal dynamics and spatial distribution of microorganisms [10,12].

This study analyzed and evaluated the inhibitory mechanism of the crude extract of substances produced by the metabolism of antagonistic bacteria against *Fusarium* causing banana wilt. The analysis was performed based on lipid peroxidation intensity, protein content, and pectinase activity of the pathogen. The whole genome of *Bacillus subtilis* was sequenced and analyzed. The genomic information was classified and analyzed by functional annotation to explore the biosynthetic pathway of the active compound. The prevention and control effect reported by the previous antifungal basic research was verified by the pot method to obtain a novel, unique, highly active, and safe biocontrol agent for controlling banana wilt.

## 2. Materials and Methods

Soil samples were collected from three sampling points around the rhizosphere of banana plants in an infected banana garden in Zhanjiang, Guangdong Province (21.2° N, 110.3° E). The samples were transferred into a self-sealing bag, thoroughly mixed, and then carried to the laboratory for further use.

The medium for initial screening was a double-layer plate to which 10 mL of sterile water was added. The pathogen was scraped into 90 mL of sterile water with glass beads. The culture was shaken at 28 °C and 170 rpm for 20 min. The bacterial solution was filtered with a double layer of sterile gauze. The spore suspension (10^5^ CFU/mL) of banana Fusarium wilt (*F. oxysporum* f. sp. *cubense Race* 4) was prepared, and 1 mL of the spore suspension was added to 250 mL of the PDA medium cooled to 45–55 °C, mixed, and poured into the plate. After the PDA plate was cooled and solidified, the beef paste cooled to 45–55 °C was poured into the plate to obtain a double-layered plate. The growth medium of the pathogen was PDA, and the purification and growth medium of antagonistic bacteria was the beef extract [13]. The fermentation medium of antagonistic bacteriostatic substances was determined using the formula of Jourdan et al.

### 2.1. Screening of Antagonists

Initial screening was performed by incubating the double-layered plates at 28 °C for 1 day before removal. The collected soil samples were subjected to a gradient dilution process, and the resulting dilutions (10^−3^–10^−6^) were spread on the double-layered plates. The strain with an antagonistic effect on the pathogen was selected by inverting the culture at 28 °C for 3–4 days [14].

Rescreening was performed using the plate confrontation test. The pathogen block was dug with a hole punch (5 mm in diameter) and placed at the center of the PDA plate. After 1 day of culture at 28 °C, the strains were tested at a point 2.5 cm around the pathogen block. After another 3–5 days of culture at 28 °C, the inhibition of the tested strains against the pathogens was observed [15].

### 2.2. Antifungal Effect of Antagonistic Fermentation Supernatant

The selected antagonists were added to a fresh fermentation medium and incubated at 30 °C for 48 h on a shaker at 170 rpm, then centrifuged at 10,000 rpm for 5 min. The supernatant was collected and filtered with a sterile membrane (diameter 0.22 μm) to obtain a sterile supernatant. The pathogen block was dug with a hole punch (5 mm in diameter) and placed at the center of the PDA plate. After 1 day of culture in an incubator at 28 °C, sterile Oxford cups were placed at 2.5 cm around the pathogen block; 200 μL of sterile supernatant was then added and cultured in an incubator at 28 °C for 4–5 days before recording the inhibition of pathogens [16].

### 2.3. Identification of Antagonists

Physiological and biochemical identification: The HBI microbial biochemical identification strip was used for the physiological and biochemical identification of antagonistic bacteria, with 15 indicators [13]. The process included experiments on single carbon source utilization, single nitrogen source utilization, and enzyme characteristics.

*16S rRNA* gene identification: The *16S rRNA* gene fragment of the strain was amplified by polymerase chain reaction (PCR), and the primer sequences were 27F: 5′-AgagTTTGATCCTGGCTCAG-3′) and 1492 r: 5′-GGTTACCTTGTTACGACTT-3′. The PCR reaction system (50 μL) included 2.5 mmol/L dNTPs, 4 μL; 10× buffer, 5 μL; 25 mmol/L MgCl_2_, 5 μL; template DNA, 1 μL; 10 μmol/L primers, 1 μL; 5 U/μL Taq enzyme, 0.5 μL; and double-steamed water, 32.5 μL. The PCR amplification conditions were as follows: 94 °C for 5 min; 94 °C for 30 s; 52 °C for 30 s; 72 °C for 1 min, 30 cycles; and 72 °C for 10 min. The PCR products were detected by agarose gel electrophoresis and then sent for sample sequencing. The sequencing results were uploaded to NCBI and GenBank for the Basic Local Alignment Search Tool (BLAST, NCBI, USA) analysis. The phylogenetic tree of the strain was constructed using MEGA 7.0.

### 2.4. Scanning Electron Microscopy Detection

The samples were pre-frozen at −40 °C for 24 h, vacuum freeze-dried, sputtered, and gold-plated. Before observation, the sample was fixed on the sample copper platform, and the vacuum degree was 1.5 × 10^−3^ Pa. The samples were observed using an electron microscope and photographed at an accelerated voltage of 5 kV. After obtaining scanning electron microscopy images of the micromorphology of samples added (20,000×), the representative areas were selected for the capturing of the images [17].

### 2.5. Controlling Effect of Antagonistic Bacteria on Banana Fusarium wilt in Greenhouse

The test soil was healthy and had never been used to grow bananas. The test entailed three treatments: CK, inoculated with *Bacillus* only; JF-4, inoculated with *Bacillus* and antagonistic bacteria JF-4; and JF-5, inoculated with pathogen and antagonistic bacteria JF-5. The root irrigation was conducted 7 days after transplantation. The initial concentration of *Bacillus* was adjusted to 1.0 × 10^5^ CFU/g dry soil. The next day, two antagonistic strains were inoculated to adjust the initial concentration to 1.0 × 10^7^ CFU/g dry soil. Ten pots were included in each group, with one seedling planted in each pot. Thirty pots were randomly arranged and placed in the greenhouse with regular water and fertilizer management. The growth and blight of bananas were observed. The yellowness and blight of the stem and leaves were observed after inoculation with pathogenic bacteria. The growth of bananas was recorded daily for the test period of 60 days from May to July 2021 [18].

### 2.6. Effects of Bactericidal Extracts on Pathogen Growth and Metabolism

Crude extraction of antagonistic bactericidal inhibitor: The supernatant from the antagonistic bactericidal fermentation was collected, and its pH was adjusted to 2.0 using 6 mol/L HCl. It was then placed at 4 °C to precipitate overnight. The pH of the supernatant was restored to its initial value of pH 7.0, and the precipitate was dissolved in pure methanol. The precipitate was incubated again at 4 °C for 4 h. After removal, the supernatant was retained by centrifuging at 8000 rpm for 20 min. The antifungal activity of the supernatant and acid precipitate was determined using the Oxford cup method [19].

Treatment of mycelial pathogens: A conidia suspension of pathogens was inoculated in 50 mL of PDA liquid medium and incubated at 28 °C for 5 days, which resulted in a final concentration of conidia of 1 × 10^7^ CFU/mL. The extracted antagonistic and antifungal substances from 1.6 L were added to a final concentration of 100 mg/L, and the mixture was incubated at 28 °C for 5 days before use [15].

### 2.7. Effects of Antagonistic Bacteria on the Related Indices of Pathogens

The effects of antagonistic bacteria on the lipid peroxidation and ergosterol levels of pathogenic fungi and the protein content and pectinase activity of pathogenic mycelia were investigated using the corresponding kit (purchased from Tiangen Biochemical Technology (Beijing) Co., Ltd., Beijing, China) [20].

The dried mycelia was ground into a powder using the saponification method, and 0.10 g was weighed and added into a mixture of 10 mL of methanol and chloroform (volume ratio of 3:1). After mixing, the mixture was extracted by ultrasound three times, 15 min each time. The extraction solution was centrifuged at 3000 rpm for 5 min, and the supernatant was taken, followed by 10 mL each of water, chloroform, and 0.5 mol/L potassium phosphate buffer containing 2.0 mol/L potassium chloride (pH 6.8). After extraction and stratification, the chloroform phase was taken and rotated to dry at 45 °C. The mixture of methanol and ethanol (volume ratio 4∶1) containing 1.4 mol/L potassium hydroxide was added to a final concentration of 10 mL and saponified at 60 °C for 1.0 h. After adding 10 mL of water and 10 mL of petroleum ether, the extracted phase of petroleum ether was distilled at 37 °C, the volume of ethanol was fixed to 10 mL, and then the mixture was filtered through a microporous organic filter membrane (pore size, 0.22 μm). The detection was performed using the UPLC external standard method, and each treatment was repeated three times.

### 2.8. Component Analysis of Extracts of JF-4 and JF-5 by Gas Chromatography–Mass Spectrometry

To obtain the active lead components in crude bacteriostatic extracts of strain JF-4 and JF-5, the strain was incubated in PDA medium in a conical flask. After incubation for 1 d at 28 °C, the active lead components were collected and determined using ahead-space solid phase micro-extraction (SPME, 57324-a) coupled with gas chromatography–mass spectrometry (GC-MS). A TG-5MS elastoplastic quartz capillary column (30 mm × 0.25 mm × 0.25 μm) was used. The initial temperature was 60 °C; after holding for 2 min, the rate was raised to 100 °C at 10 °C/min, then raised to 120 °C at 6 °C/min, and then raised to 180 °C at 2 °C/min. The partial flow ratio was 20:1, and the flow rate was 1.0 mL/min. electron energy 70 ev, ion source temperature 200 °C, interface temperature 290 °C, and scanning range 30–500 amu [21].

### 2.9. Genomic DNA Extraction of B. subtilis JF-4

*B. subtilis* JF-4 was inoculated in the ISP2 liquid medium at 28 °C for 3 days at 180 rpm under shock and then centrifuged at 4 °C for 10 min at 10,000 rpm. The supernatant was poured out, and the bacteria were collected and frozen in liquid nitrogen. Genomic DNA was extracted using a QIAGEN Genomic Genome-Tip kit (Guangzhou Jianlun Biotechnology Co., LTD, Guangdong, China) [20].

### 2.10. B. subtilis JF-4 Gene Library

The DNA was sent to Beijing Baimaike Biotechnology Co., Ltd. (Beijing, China) for whole-genome sequencing. After passing the quality inspection, the genomic DNA was randomly interrupted and the magnetic beads were used to enrich, purify, and gel large DNA fragments for recovery. Damage repair, terminal repair, and 3′ terminal addition of A were performed on the screened large fragments of DNA. The repair products were connected and purified to obtain the final computer library. A certain amount of DNA library was taken, mixed with related reagents on the computer, and added into the flow cell. The real-time single-molecule sequencing technology was used to obtain the original sequencing data [17].

### 2.11. Sequencing and Assembly of B. subtilis JF-4

The filtered subreads were assembled using the Canu v1.5/wtdbg v2.2 software. The Pilon v1.22 software was used to further correct the assembled genome with the second-generation data, and the final genome with higher accuracy was obtained. The assembled contig sequence was compared with the NT database to determine the chromosome type. The chromosome sequence was assembled into a circular gene group [12].

### 2.12. Functional Annotation of B. subtilis JF-4 Genome

The predicted gene sequences were compared with *Clusters of Orthologous Groups* (COG), *Kyoto Encyclopedia of Genes and Genomes* (KEGG), *Swiss-Prot*, *TrEMBL*, *Nr*, and other functional databases in the BLAST v2.2.29 software to obtain the gene functional annotation results. Based on the comparison results of the *Nr* database, the Blast2GO v2.5 software was used to annotate the function of the *Gene Ontology* (GO) database. The software hmmer v3.0 was used to annotate Pfam functions based on the *Pfam* (27.0) database. In addition, the COG, KEGG metabolic pathway enrichment analysis, GO functional enrichment analysis, and other gene functional annotation analyses were performed [11].

### 2.13. Statistical Analyses

All data were exported and processed by the Microsoft Excel program and were analyzed using the IMB SPSS version 22. All data were presented as mean values with their standard deviations (mean ± standard deviation, *n* = 3). At a 95% confidence level, ANOVA and LSD multiple comparison tests were used for statistical analyses. * *p* < 0.05 and ** *p* < 0.01 indicated statistically significant differences.

## 3. Results

### 3.1. Isolation and Screening of Antagonists

**Figure 1:**
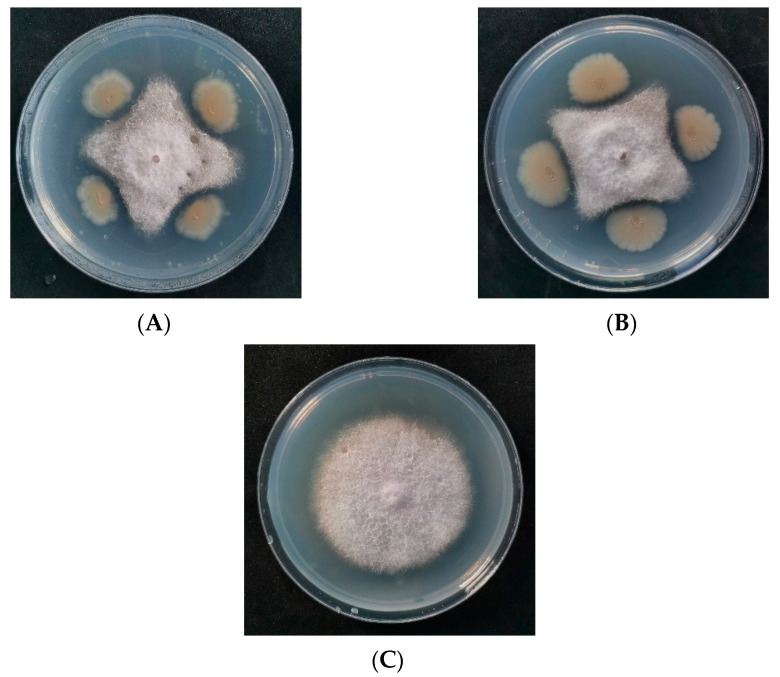
Inhibition of *Fusarium oxysporum f.* sp. *cubense* of strains JF-4 and JF-5. Note: (**A**,**B**) indicate the antagonism effects against pathogen showed by strain JF-4 and JF-5 on the plates, respectively; (**C**) is indicated as control.

After the double-plate screening, six strains of bacteria produced a bacteriostatic zone around the pathogen. After selecting the single colonies and crossing them repeatedly, the colonies were determined according to their shape. Four antagonistic strains were selected for double screening (Supplementary Figure S1). After determining the antagonistic effect of the fermentation supernatant of four strains, the antagonistic bacteria labeled JF-4 and JF-5 exhibited inhibitory effects against the pathogen (Figure 1).

### 3.2. Physiology and Biochemistry of Antagonists and Identification of the 16S rRNA Gene

**Figure 2:**
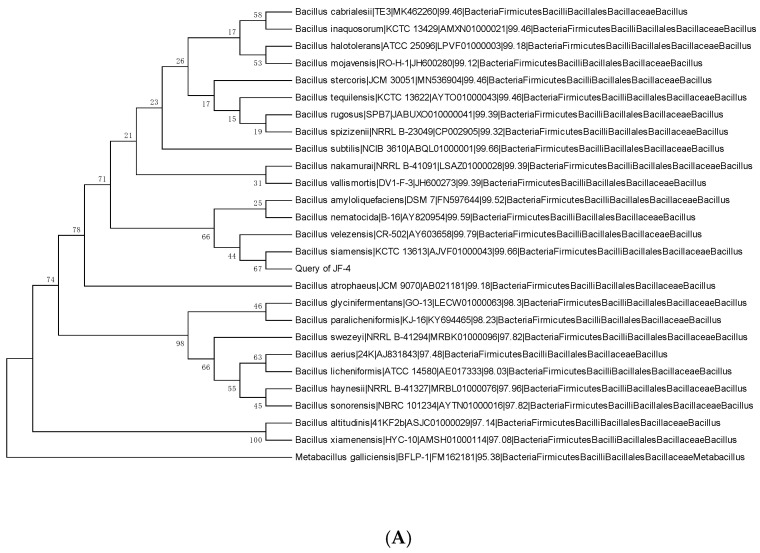

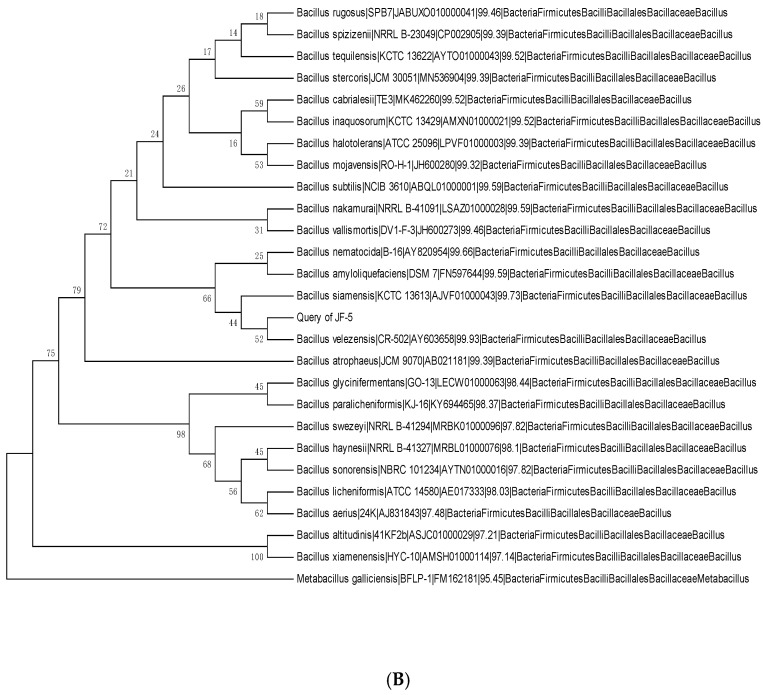
Phylogenetic trees based on the partial 16S rRNA gene sequence of JF-4 (**A**) and JF-5 (**B**). Note: Numbers on the branch point indicate bootstrap value of the branch; Numbers in the parentheses indicate GenBank accession number of the strain; Scale plate on the figure indicates evolutionary distance.

Based on the physiological and biochemical indicators, antagonistic bacteria were identified as Gram-positive bacteria. They tested negative for hydrogen sulfide, urease, mannitol, and sorbitol, and positive for other indicators (Table 1). The *16S rRNA* gene amplification products of antagonistic bacterial strains JF-4 and JF-5 were sequenced. The information was uploaded to NCBI for alignment, and sequences with high similarity were obtained. The phylogenetic tree was constructed using the neighbor-joining method in the MEGA 6.0 software. After identification (Figure 2), the antagonistic bacteria (JF-4) and *B. subtilis* were in the same branch, and the similarity of the NCBI comparison was 99% (Figure 2A). The antagonistic bacterium JF-5 was closest to the branch of *B. amylum*, based on the NCBI comparison, with a 99% similarity (Figure 2B).

### 3.3. Amplification of Antagonistic-Specific Primers

**Figure 3:**
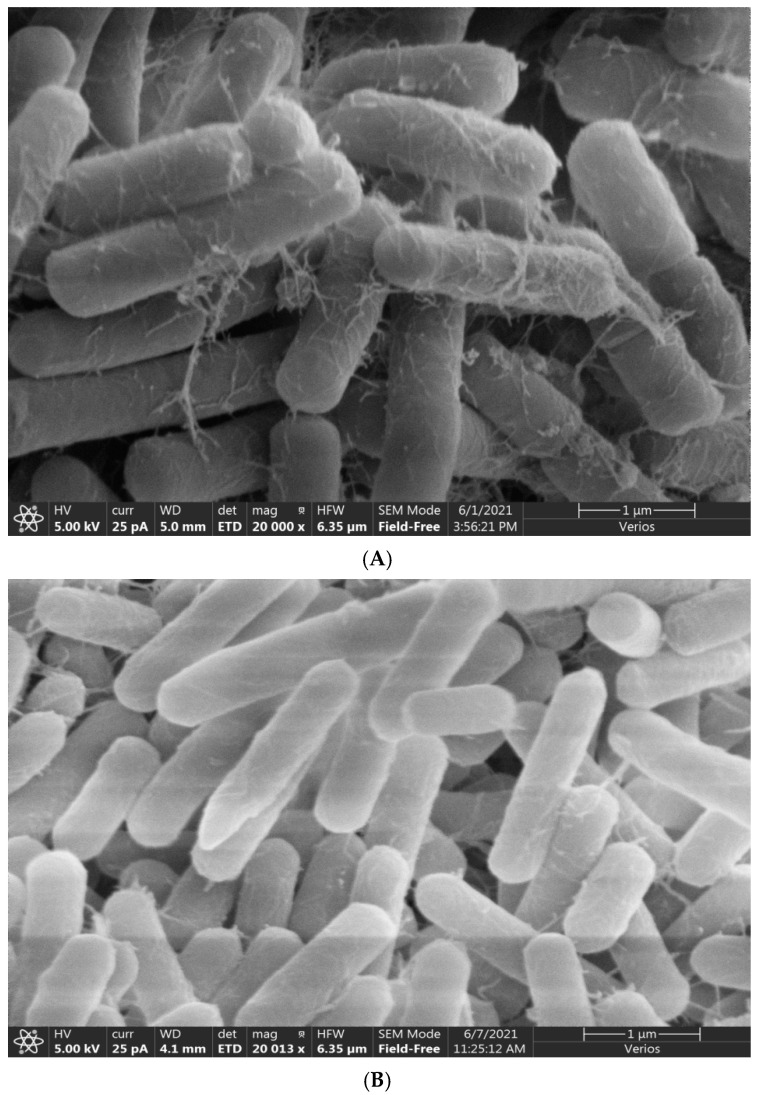
Scanning electron micrographs of strains JF-4 (**A**) and JF-5 (**B**).

Combined with the previous identification results, the antagonistic bacteria JF-4 and JF-5 were identified as *B. subtilis* and *B. amylum*, respectively. The results of transmission electron microscopy showed that the filaments of strains JF-4 (Figure 3A) and JF-5 (Figure 3B) were straight and flexible, and the morphology observed was bacillar (Figure 3).

### 3.4. Prevention and Control of Antagonistic Bacteria in Greenhouse

**Figure 4:**
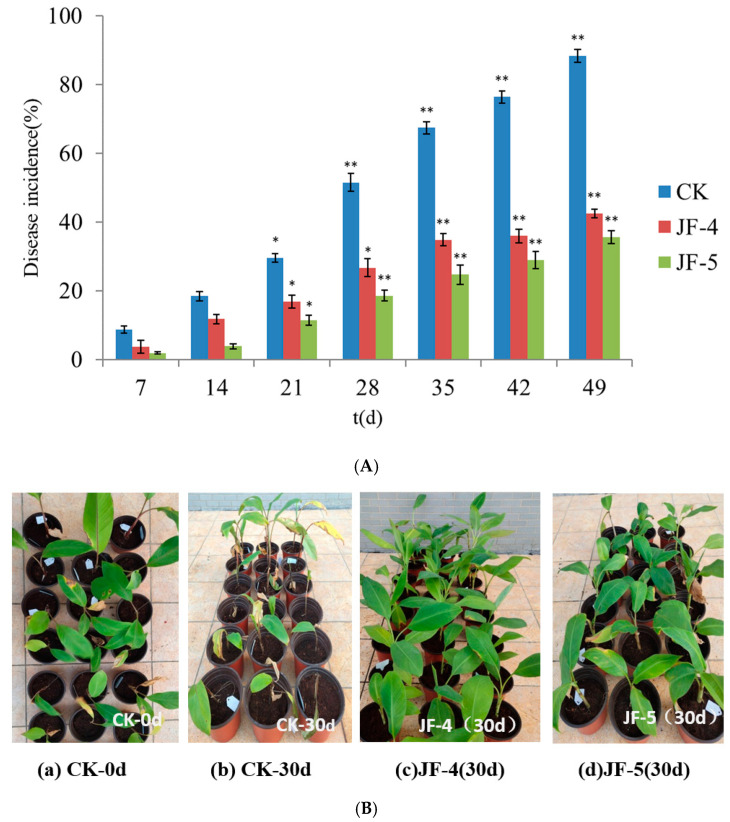
Biocontrol of Fusarium wilt disease in banana by JF-4 and JF-5. Note: (**A**) displays the disease incidence of banana seedlings under different treatments; (**B**) displays the pathological symptoms of banana seedlings under different treatments. * indicates that the difference is significant at the 0.05 level; ** indicates that the difference is significant at the 0.01 level.

Inoculation of the antagonists JF-4 and JF-5 effectively reduced the incidence of banana plants (Figure 4). The pot experiment lasted for almost 60 days. After 2 weeks of treatment, the banana began to show the disease symptoms. At the end of the experiment, the disease incidence in the CK treatment group reached 88.3%. The disease incidence in the JF-4 and JF-5 treatment groups was 42.5% and 35.6%, respectively, which was significantly lower than that in the CK group (*p* < 0.05). The biological control efficiency was 48.3% and 40.3%, respectively. In addition, the disease incidence in the JF-4 treatment group was significantly lower than that in the JF-5 treatment group throughout the experiment (*p* < 0.05).

### 3.5. Effect of Antagonistic JF-4 and JF-5 Extracts on the Cell Membrane of Foc TR4

#### 3.5.1. The Effect of CAT Content

**Figure 5:**
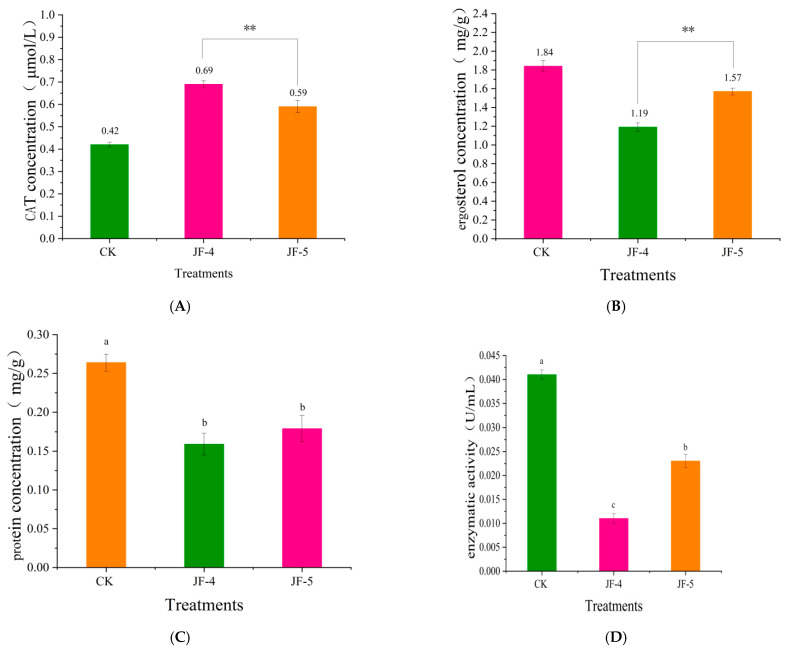
Effect of antagonistic extract on cell membrane of Foc TR4: (**A**) the concentration of CAT; (**B**) ergosterol activities; (**C**) the concentration of protein; and (**D**) the concentration of pectinase. Different lowercase letters (a, b, c) indicate that the difference is significant at the 0.05 level. “**” indicate that the difference is extremely significant at the 0.01 level.

After treatment with crude extracts of antagonistic bacteria JF-4 and JF-5, the catalase (CAT) level in the mycelia of the pathogen was significantly higher than that in the control group (*p* < 0.05) (Figure 5A). The effect of the antagonist JF-4 was stronger and significantly greater than that of JF-5. The effects of both were significantly greater than that in the control group, with a CAT level of 0.42 μmol/L. CAT represents the peroxidation of cell membrane lipids. High and low levels indicate the extent of cell membrane damage. The results demonstrate that the antifungal substances of antagonists JF-4 and JF-5 significantly damaged the cell membrane of the pathogen, a process which might be mediated via antagonistic antifungal inhibition.

#### 3.5.2. Effects of Ergosterol Content in Pathogenic Fungi

Treatment with crude antifungal extracts JF-4 and JF-5 resulted in a significant decrease in the ergosterol activity of pathogenic fungi (*p* < 0.05) (Figure 5B). The rate of decrease was higher in the JF-4 treatment group than in the JF-5 treatment group. At the end of the experiment, the level of ergosterol in the control group was 1.84 mg/g, compared with 1.19 and 1.56 mg/g in the JF-4 and JF-5 treatment groups (*p* < 0.05), respectively.

#### 3.5.3. Changes in Soluble Protein Content

The antagonistic antifungal substances JF-4 and JF-5 significantly reduced the protein content of pathogenic fungi (*p* < 0.05) (Figure 5C). At the end of the experiment, the protein concentration of the pathogen in the control group was 0.264 mg/g, compared with 0.159 mg/g and 0.177 mg/g in the samples treated with JF-4 and JF-5 (*p* < 0.05), respectively. Protein is the product of metabolism in the somatic cells of a pathogen. The reduction in protein synthesis indicated the weakened metabolic activity of the pathogen. The protein levels of JF-4 and JF-5 bacteria significantly reduced, indicating that the bacteriostatic extracts affected the metabolism of the pathogen, thereby affecting the growth of bacteria.

#### 3.5.4. Effect of the Pectinase Levels

After the treatment with crude bacteriostatic extracts JF-4 and JF-5, the pectinase activity of the pathogen significantly decreased (*p* < 0.05) (Figure 5D). The lowest activity of JF-4 treatment was associated with a pectinase concentration of 0.0011 U/mL. The activity of the JF-5 treatment was 0.023 U/mL, which was significantly higher than that of the JF-4 treatment (*p* < 0.05); the highest value was 0.041 U/mL, following the CK treatment.

### 3.6. Analysis of Metabolic Components of Strain JF-4 and JF-5 Extracts Using GC–MS

The metabolic components produced by antagonistic bacteria JF-4 and JF-5 were analyzed by GC-MS. Twenty chemical compounds were identified by the comparison of their mass spectra with the NIST library, based on retention time, molecular mass, and molecular formula; their chemical structures are shown in Table 1. The emission spectrum showed that the extracts of antagonistic bacteria JF-4 and JF-5 mainly contained hydrocarbons, acids, terpene, and other components (Table 2). Among these high levels of substances, 7-methyl-Z-tetradecene-1-ol acetate, methyl ester, hexadecanoic acid, 13-docosadecamide, and tetradecane possessed antifungal, antimicrobial, insecticidal, and antitumor activities [21,22,23,24,25].

### 3.7. Genomic Characterization of B. subtilis JF-4

**Figure 6:**
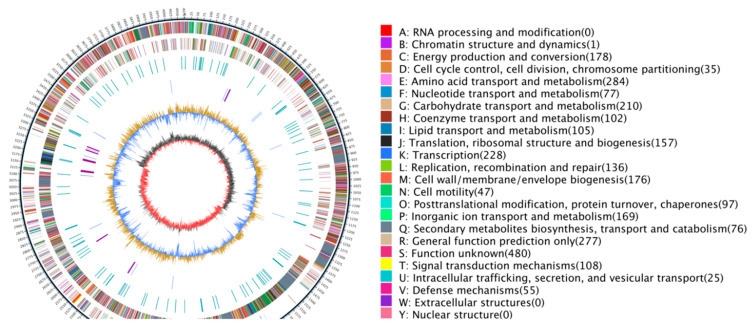
Circular graph of the *B. subtilis* JF-4 genome. Note: The outer scale is numbered in intervals of 0.5 Mb from the left to the right ends. Circle 1 in the solid line shows the core (blue) and arms (yellow); in Circles 2 and 3 (forward and reverse strands), the predicted protein coding regions are colored according to the COG function categories; Circles 4 and 5 show the DraI and AseI sites; in Circle 6, the distribution of secondary metabolic gene clusters is colored to show different product groups, and the pink blocks indicate a region that could be recognized as a genomic island; in Circle 7, the distribution of tRNAs (light blue) and rRNA operons (red) is shown; Circle 8 shows recombinases, transposes and integrases; Circle 9 shows the CRISPR array (pink) and Cas proteins (blue); Circle 10 indicates the GC content; Circle 11 presents the GC bias. Ori, origin of replication.

*B. subtilis* JF-4 was sequenced and assembled. The genome map of *B. subtilis* JF-4 showed that 117,186 gene sequences were obtained. The genome consisted of a complete circular chromosome of 1,486,274,400 bp, and the length of N50 was 21,608 bp. The sequence was consistent with that of *B. subtilis*. When the genome was assembled, it consisted of a scaffold with 4,078,417 bp and a high G + C content of 46.34%, which was similar to the scaffold of *Actinomyces* with a high G + C content (Figure 6 and Table 3).

#### 3.7.1. *B. subtilis* JF-4 Genome Functional Annotation

A total of 6199 protein-coding genes were annotated in the *B. subtilis* JF-4 genome, accounting for 97.45% of the open reading frame in the whole genome. Unknown and unannotated sequences were still found in the whole genome. The coding genes were analyzed, predicted, and annotated to explore the different functions of protein-coding genes.

#### 3.7.2. GO Database Functional Annotation

**Figure 7:**
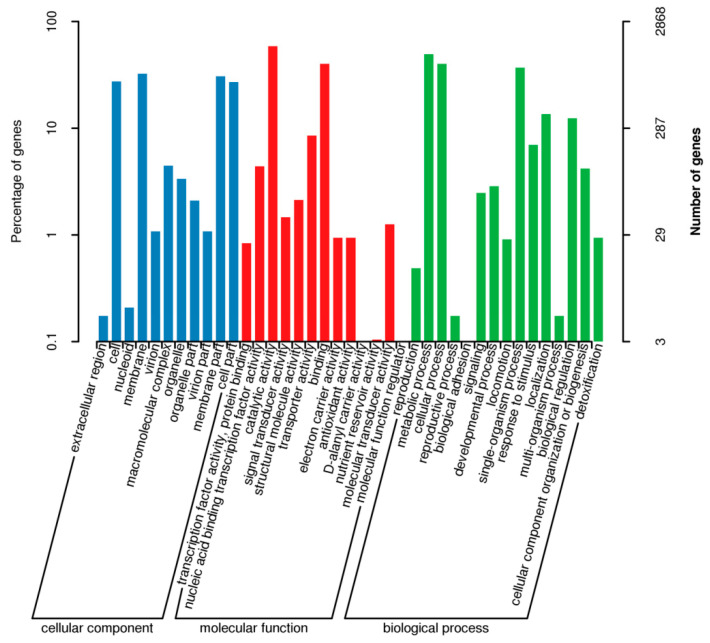
Classification map of genome of *B. subtilis* JF-4 in GO. Note: The horizontal coordinate is the content of GO classification, the left side of the vertical coordinate is the percentage of the number of genes, and the right side is the number of genes.

Among the seven GO secondary functional categories in cytology components, which are annotated to cells and cell parts, the number of protein-coding genes in the functional category was found to be the highest. However, only a few protein-coding genes were annotated in the functional categories of the extracellular region and the extracellular region part. A total of 2868 protein-coding genes of *B. subtilis* JF-4 were GO-annotated and classified into 28 functional groups, of which 1034 genes were involved in the composition of cell components (Figure 7). Then, 6045 genes were determined to be related to molecular function, and 6235 genes were involved in biological processes, making up the largest proportion. In the biological processes, 1763 genes were involved in metabolic processes. Therefore, the protein product encoded by *B. subtilis* JF-4 was mainly involved in biological processes, some of which were related to metabolic synthesis and some others were responsible for forming cellular components.

#### 3.7.3. COG Database Functional Notes

**Figure 8:**
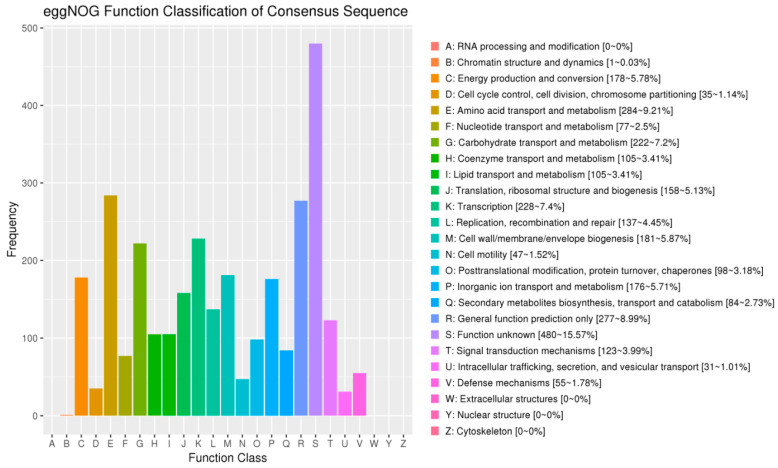
Classification map of the genome of *B. subtilis* JF-4 in COG. Note: The horizontal coordinate is the classification content of COG, and the vertical coordinate is the number of genes.

The predicted protein sequences of 4945 coding genes in the *B. subtilis* JF-4 genome were compared, using the BLAST analysis, with the protein sequences in the COG database. A total of 3867 protein-coding genes were functionally annotated using COG, and 25 functional classifications were performed. The four COG functional categories with the largest number of protein-coding genes annotated included amino acid transport and metabolism (284, 7.3%), general function prediction only (277, 7.2%), carbohydrate transport and metabolism (222, 5.7%), and cell wall/membrane/envelope biogenesis (181, 4.7%). Chromatin structure and dynamics (1, 0.02%) was the functional category of COG with the least number of protein-coding genes annotated (Figure 8).

#### 3.7.4. *Nr* Database Functional Notes

**Figure 9:**
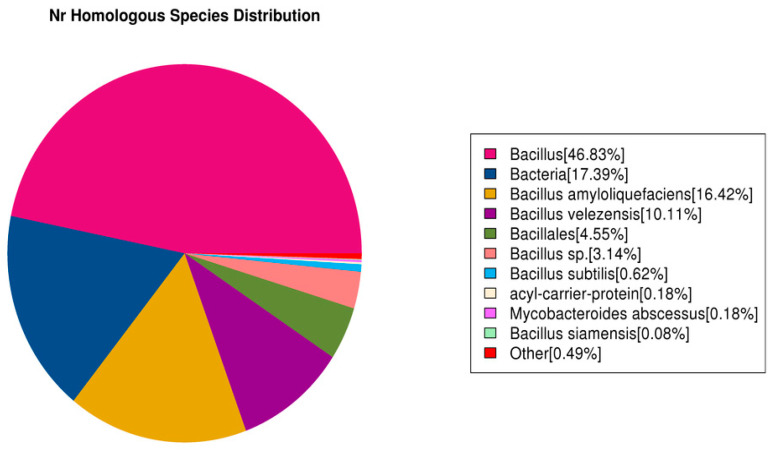
*Nr* homologous species distribution of *B. subtilis* JF-4.

Compared with other databases, the *Nr* database generally enables more genes in the genome to have annotation information. It has the highest annotation ratio. This study found that 5896 genes in the *B. subtilis* JF-4 genome were annotated in the *Nr* database, of which 46.83% were homologous to *Bacillus* (Figure 9).

#### 3.7.5. Expression of Key Genes of Secondary Metabolites of *B. subtilis* JF-4

A face-off interaction between *B. subtilis* JF-4 strain and Foc was used to identify the key antimicrobial secondary metabolites of the *B. subtilis* JF-4 strain, and the gene expression level was detected by quantitative reverse transcription (qRT)-PCR. The expression of three fengycin key synthetic genes in *B. subtilis* JF-4 increased significantly after 24 h and 36 h confrontation and interaction on the plate. Surfactin, bacillaene, and difficidin showed no significant changes in expression levels. These results suggested that fengycin might be the key antibacterial substance of *B. subtilis* JF-4.

## 4. Discussion

Both strains and fermentation supernatant antagonized the pathogen of banana Fusarium wilt Foc.4. Antagonistic bacteria JF-4 and JF-5, in greenhouse pot experiments, both exhibited specific biological control on banana Fusarium wilt. They were identified as *B. subtilis* and *B*. *amylum*. Both belonged to the genus *Bacillus*. Many biocontrol bacteria belonging to this genus have been reported. Two bacterial strains, *B. subtilis* BLG01 and BDF11, effectively controlled banana Fusarium wilt to up to 70.4% and 77.2%, respectively. Nigris et al. (2018) identified a strain of *B. amyloliquefaciens* BEB33 from the root of healthy bananas which positively affected banana Fusarium wilt [23].

A large number of studies showed that one of the mechanisms of resistance to pathogens was mediated via the production of antimicrobial metabolites. Bauer et al. (2016) screened a starchy spore stalk from saline–alkali soil. The fermentation supernatant of strain LX1 contained antifungal protein and was identified by mass spectrometry [6]. The results showed that the antimicrobial protein had the highest homology with endoglucanase designated as *B. amyloliquefaciens* FZB42. Ben Abdallah et al. (2018) isolated *B. amyloliticus* HN011 from banana wilt and other fungal diseases. It showed an antagonistic effect mediated via cyclic dipeptides against the pathogen [7]. However, the toxicological effect of the antagonist on the pathogen has rarely been reported [24,25,26,27]. The effects of antagonistic bacteria on lipid peroxidation, ergosterol, protein content, and pectinase activity in the mycelia of the pathogen were investigated to determine the bacteriostatic mechanism of the two antagonistic strains [28,29,30].

The antagonistic bacteria JF-5 had an obvious controlling effect on banana wilt because it could produce a variety of antibacterial metabolic substances and effectively inhibit the growth of pathogenic bacteria [16,20]; the best controlling effect was 65.36%. Also, JF-5 could produce hormones that promoted or improved the growth of host plants, alleviated disease symptoms caused by plant pathogens or various environmental stresses, and enhanced plant autoimmunity [11,22].

Antagonistic bacteria are known to emit VOCs with various antifungal components [26]. In this study, the antifungal ability of f JF-4 and JF-5 a VOCs against Foc TR4 was detected by double plate analysis, and it was found that it could effectively inhibit the growth of Foc TR4. Upon further investigation of the antifungal mechanism of strains JF-4 and JF-5′s VOCs against Foc TR4, it was found that the main components were acids, terpenes, and hydrocarbons. The highest content of 7-methyl-Z-tetradecene-1-ol acetate was in the VOCs. The 7-methyl-Z-tetradecene-1-ol acetate was proven to be responsible for the inhibitory activity against *Bacillus* [31]. This indicated that VOCs produced by actinomycetes were rich in antifungal substances which functioned to inhibit the growth of phytopathogenic fungi.

Actinomycetes inhibited pathogenic bacteria mainly by producing active secondary metabolites that destroyed the cell wall, cell membrane, protein synthesis system, and energy metabolism process of mycelia. Antagonists JF-4 and JF-5 both significantly increased the CAT levels in mycelia and reduced the levels of ergosterol, protein, and pectinase. The increased CAT level and the decreased ergosterol content reflected the extent of bacterial damage to the membrane structure of the filament [3,15]. Leger et al. (2021) reported the mechanism of antifungal substances antagonizing the cucumber Fusarium wilt pathogen [17]. The levels of propylene II, aldehyde, and ergosterol in the tested pathogen hyphae increased, and then decreased gradually with the increasing concentrations of bacteriostatic substances. The degree of membrane damage increased. Our results were similar, suggesting that the mycelial membrane of the pathogen treated with two antagonistic strains was seriously damaged, resulting in the inhibition of the pathogen growth, which was one of the antifungal mechanisms of JF-4 and JF-5.

Some studies have pointed out that ergosterol is the pathogenic factor in the pathogen [30,31,32,33]. Inhibition of the activity of ergosterol can partially reduce the virulence of the pathogen, and the bacteriostatic effect can be achieved. Our results showed that the antagonistic bacteria JF-4 and JF-5 significantly inhibited the ergosterol activity of the pathogen, indicating that the two strains decreased the virulence of pathogenic fungi, which might be one of the antifungal mechanisms. Pectinase is a pathogenic factor secreted by pathogenic bacteria. Inhibition of pectinase activity can partially decrease the pathogenicity of the pathogen, resulting in a bacteriostatic effect. Our results showed that the antagonists JF-4 and JF-5 significantly inhibited the pectinase activity of the pathogen, indicating that these two strains reduced the pathogenicity of the pathogen, which might be one of the bacteriostatic mechanisms.

The effect of the pathogen on the cellular metabolites can be determined partially by analyzing the effect of antagonistic bacteria on the protein content of pathogenic mycelia. Our results showed that the protein content of mycelia was significantly reduced by the antagonistic treatment, which differed significantly from that of the control treatment, indicating that the intracellular metabolism of the pathogen was blocked, and the growth of the bacteria was affected. The results were consistent with those of Moore et al. (2019). Further, pectinase promoted the induction of virulence in banana Fusarium wilt. Thus, the metabolism of the antagonists JF-4 and JF-5 had a significant inhibitory effect on the pectinase synthesized by banana Fusarium wilt, which significantly decreased the virulence of the pathogen, resulting in an antifungal effect. Moore et al. (2021) also reported that the antifungal compounds of *Sophora flavescens*, an endophytic fungus, reduced the pathogenicity of *P. tomatidis* by inhibiting its extracellular enzyme activity [21].

Surfactin, bacillaene, fengycin, and difficidin were used to analyze secondary metabolite synthesis gene clusters based on *B. subtilis*. Nigris et al. (2018) analyzed the whole-gene sequence of *B. subtilis* JF-2. These strains have been associated with the synthesis of various NRPS (bacillibactin, bacilysin, fengycin, and surfactin) and antimicrobial PKS (bacillaene, diffificidin, and macrolactin), all of which have direct or indirect antifungal and bacterial activities. The expression levels of key synthetic genes of the secondary metabolite of *Bacillus subtilis* JF-4 were analyzed. Three key synthetic genes of fengycin were highly expressed when *B. subtilis* JF-4 interacted with Foc antagonism, suggesting that fengycin might be the main secondary metabolite of the *Bacillus* strain involved in inhibiting banana Fusarium wilt. Liu et al. (2020) showed that *Bacillus* HC6 produced an antifungal metabolite named fengycin that showed obvious antifungal activity against *Aspergillus* and *Fusarium*. Xiong et al. (2015) isolated *B. amyloliquefaciens* JK6 from the rhizosphere soil of tomato plants and identified the antifungal metabolite as fengycin [17]. Therefore, fengycin may be a biocontrol substance of *Bacillus subtilis* which functions to protect plants from pathogen invasion, and it might have a remarkable antifungal effect on plant pathogenic fungal diseases.

Genomic analysis is of great significance to the diversity, environmental adaptability, and application of various bioactive substances of actinomycetes. *B. subtilis* JF-4 genome sequence analysis showed that its genomic length was 681,804,824 bp, and the (G + C) mol% content was 68.45%. In this study, the COG database showed that 3867 genes were involved in the synthesis, transport, and metabolism of *B. subtilis* JF-4 secondary metabolites, accounting for 78.2% of the total number of genes annotated. The predictive analysis of anti-SMASH software showed that *B. subtilis* JF-4 could produce abundant secondary metabolites. This provides a new perspective for understanding the mechanism by which *B. subtilis* JF-4 may prevent disease and promote growth, and is of great significance for future research on *B. subtilis* JF-4.

## 5. Conclusions

The crude extract of antagonistic bacterial strains JF-4 and JF-5 inhibited the pectinase activity and protein levels of banana Fusarium wilt by disrupting the cell membranes. Therefore, these antagonistic bacteria, JF-4 and JF-5, can be used as biological control agents to inhibit pathogenic fungi and promote the growth of bananas. Twenty compounds were identified by GC-MS. *B. subtilis* JF-4 was sequenced and assembled to obtain a complete circular chromosome genome of 681,804,824 bp. The genome consisted of a 4,310,825-bp-long scaffold. The content of G + C in the genome was 68.45%.

## Figures and Tables

**Table 1:** Physiological and biochemical characteristics of antagonists JF-4 and JF-5.

Characteristics	JF-4	JF-5
Citrate	+	+
Hydrogen sulfide	−	−
Urease	−	−
Lactose	+	+
Gelatine	+	+
Glucose	+	+
Arabinose	+	+
Gram stain	+	+

Note: +: Positive effect; −: Negative effect.

**Table 2:** Components identified by GC-MS from JF-4 and JF-5 crude extract.

Retention Time	Molecular Formula	Molecular Mass	Possible Compound Classification	
3.45	C_6_H_12_O_2_	116	Methylguanidine	
4.30	C_9_H_16_ClN_5_	229	Phenylacetamide, Others	
11.38	C_12_H_20_F_3_NO_3_	283	3-Furancarboxylic acid, methyl ester	
15.33	C_5_H_4_N_4_O_3_	168	2,4-bis (1,1-dimethylethyl)-phenol	
2.23	C_14_H_22_O	206	2-hydroxypropanediamine acid dimethyl	
6.71	C_7_H_10_N_2_O_2_	154	diethyl phthalate	0.71
9.32	C_16_H_22_ClNO	279	methyl ester, Hydrocarbons	0.32
17.96	C_8_H_9_NO	135	2,4-decadienal	1.96
20.43	C_27_H_54_	378	7-methyl-Z-tetradecene-1-ol acetate	
26.44	C_18_H_36_O_2_	284	dimethyltetradecan-1-thiol	
30.32	CH_3_(CH_2_)_12_CH_3_	198	Tetradecane	
4.89	C_8_H_8_O_2_	136	2-phenylacetic acid	
8.42	C_18_H_36_O_2_	284	hexadecanoic acid	
25.67	C_16_H_32_O_2_	256	hexadecanoic acid, Acids	
26.01	C_18_H_36_O_2_	284	9,12-octadecadienoic acid	
35.11	C_16_H_32_O_2_	256	oleic acid	
10.48	C_17_H_34_O_2_	270	pyrazine-1,4-dione	
22.44	C_19_H_38_O_2_	298	2-diphthalic acid isooctyl esters	
31.87	C_21_H_38_O_2_	322	13-docosadecamide, Terpene	
42.64	C_20_H_36_O_2_	308	Guaia-10(14),11-diene	

**Table 3:** Genomic sequence clean data of *B. subtilis* JF-4.

Genome		Genome Assembly	
Number of sequences	117,186	Scaffold length (bp)	4,078,417
Number of bases (bp)	1,486,274,400	Scaffold number	1
N50 reads length (bp)	21,608	Scaffold N50 (bp)	4,078,417
N90 reads length (bp)	5366	Scaffold N90 (bp)	4,078,417
Mean reads length (bp)	12,683	GC content (%)	46.34
Mean reads quality	12.21	Gaps number	0

## Data Availability

Not applicable.

## Supplementary Materials

The following supporting information can be downloaded at: https://www.mdpi.com/article/10.3390/jof9090886/s1, Figure S1: Double screening of antagonistic strains.
